# Aging increases microglial proliferation, delays cell migration, and decreases cortical neurogenesis after focal cerebral ischemia

**DOI:** 10.1186/s12974-015-0314-8

**Published:** 2015-05-10

**Authors:** Ana Moraga, Jesús M Pradillo, Alicia García-Culebras, Sara Palma-Tortosa, Ivan Ballesteros, Macarena Hernández-Jiménez, María A Moro, Ignacio Lizasoain

**Affiliations:** Unidad de Investigación Neurovascular, Departamento de Farmacología, Facultad de Medicina, Universidad Complutense, Avda. Complutense s/n, 28040 Madrid, Spain

**Keywords:** Interneurons, Microglia, Neuroblast, Neutrophil, Proliferation, Stroke

## Abstract

**Background:**

Aging is not just a risk factor of stroke, but it has also been associated with poor recovery. It is known that stroke-induced neurogenesis is reduced but maintained in the aged brain. However, there is no consensus on how neurogenesis is affected after stroke in aged animals. Our objective is to determine the role of aging on the process of neurogenesis after stroke.

**Methods:**

We have studied neurogenesis by analyzing proliferation, migration, and formation of new neurons, as well as inflammatory parameters, in a model of cerebral ischemia induced by permanent occlusion of the middle cerebral artery in young- (2 to 3 months) and middle-aged mice (13 to 14 months).

**Results:**

Aging increased both microglial proliferation, as shown by a higher number of BrdU^+^ cells and BrdU/Iba1^+^ cells in the ischemic boundary and neutrophil infiltration. Interestingly, aging increased the number of M1 monocytes and N1 neutrophils, consistent with pro-inflammatory phenotypes when compared with the alternative M2 and N2 phenotypes. Aging also inhibited (subventricular zone) SVZ cell proliferation by decreasing both the number of astrocyte-like type-B (prominin-1^+^/epidermal growth factor receptor (EGFR)^+^/nestin^+^/glial fibrillary acidic protein (GFAP)^+^ cells) and type-C cells (prominin-1^+^/EGFR^+^/nestin^−^/Mash1^+^ cells), and not affecting apoptosis, 1 day after stroke. Aging also inhibited migration of neuroblasts (DCX^+^ cells), as indicated by an accumulation of neuroblasts at migratory zones 14 days after injury; consistently, aged mice presented a smaller number of differentiated interneurons (NeuN^+^/BrdU^+^ and GAD67^+^ cells) in the peri-infarct cortical area 14 days after stroke.

**Conclusions:**

Our data confirm that stroke-induced neurogenesis is maintained but reduced in aged animals. Importantly, we now demonstrate that aging not only inhibits proliferation of specific SVZ cell subtypes but also blocks migration of neuroblasts to the damaged area and decreases the number of new interneurons in the cortical peri-infarct area. Thus, our results highlight the importance of using aged animals for translation to clinical studies.

## Background

Stroke remains a colossal therapeutic challenge. Being a leading cause of death and disability worldwide, the only treatment available in the acute phase of stroke is thrombolysis with tissue plasminogen activator (tPA), a drug that can be administered to a low percentage of patients due to its narrow therapeutic window (<4.5 h) and safety concerns. In contrast, in the chronic phase, a broader window might exist for therapeutic interventions that may serve to promote repair in late stages and to decrease stroke-associated disability.

Importantly, stroke affects predominantly the elderly, a population segment that is dramatically rising due to the increased life expectancy. But old age is not just a risk factor of stroke: it has also been associated with an enhanced susceptibility to stroke and poor recovery from brain injury (for review, see [1]). In spite of this, most research is developed in young animals, whereas the effect of aging remains understudied.

Several reasons may account for the increased susceptibility of the aged brain. For instance, aged brain is characterized by the existence of an activated basal state of low-grade chronic inflammation which has been called ‘*inflamm-aging*’ [2] and which might lead not only to more severe and persistent behavioral and cognitive deficits after stroke but also to an impaired recovery during the chronic phase of stroke [3-5]. In addition, aging is also associated with microgliosis, a cellular response that may have both beneficial and deleterious consequences for neurogenesis (for review, see [6]). In this line, it is widely accepted that adult neurogenesis is decreased but maintained in the aging brain after injury (for review, see [1]). However, how neurogenesis is affected by aging, a situation in which infarct size, microglial, and inflammatory response are likely to be affected, is still unclear and controversial.

Therefore, the objective of this study is to explore further the role of aging on the process of neurogenesis after stroke by analyzing proliferation, migration, and formation of new neurons in young- (2 to 3 months) and middle-aged (13 to 14 months) mice exposed to focal cerebral ischemia by a permanent middle cerebral artery occlusion (MCAO).

## Methods

### Animals

Adult male C57BL/10 J young (2 to 3 months) and aged (13 to 14 months) mice were used (Jackson Labs, Bar Harbor, ME, USA). All experimental protocols adhered to the guidelines of the Animal Welfare Committee of the Universidad Complutense (EU directives 86/609/CEE and 2003/65/CE). Mice were housed under standard conditions of temperature and humidity and a 12-h-light/dark cycle (lights on at 08:00) with free access to food and water.

### Experimental groups

All groups were performed and quantified in a randomized fashion (coin toss) by investigators blinded to each specific treatment. Mice were subjected to a distal permanent middle cerebral artery occlusion (MCAO) by ligation. Sham-operated animals, considered as control group, were subjected to anesthesia and the surgical procedure, but the occlusion of the artery was omitted.

5-Bromo-2′-deoxyuridine (BrdU, 50 mg/kg) was injected i.p. once daily from days 5 to 6 after ischemia. Cell proliferation was quantified 7 and 14 days after ischemia by immunohistochemical studies.

### Induction of permanent focal ischemia

Surgery leading to focal cerebral ischemia was conducted under anesthesia with isoflurane in a mix of O_2_ and N_2_O (0.2/0.8 L/min). During surgery, body temperature was maintained at 37.0°C ± 0.5°C using a servo-controlled rectal probe-heating pad. The surgical procedure was a variant of that described by Chen *et al*. [7]. A small craniotomy was made over the trunk of the left middle cerebral artery and above the rhinal fissure. The permanent middle cerebral artery (MCA) occlusion (MCAO) was done by ligature of the trunk (proximal occlusion) just before its bifurcation between the frontal and parietal branches with a 9-0 suture. Because we demonstrated that cell proliferation is dependent on infarct size after stroke [8] and because there is also contradictory information in the literature on the effect of age on infarct size, we included an additional group in which MCAO was performed on a posterior branch of the MCA (distal occlusion) to obtain smaller infarct volumes. The method used is exactly the same for both, the only difference being the site in which the occlusion is performed. Complete interruption of blood flow was confirmed under an operating microscope.

Physiological parameters (rectal temperature, mean arterial pressure, pO_2_, pCO_2_, pH) were not significantly different between all studied groups (data not shown). No spontaneous mortality was found after MCAO with this model, and this was unaffected by the different experimental groups.

### Infarct size determination

For infarct size determination 48 hours after MCAO, magnetic resonance examination was performed using a BIOSPEC BMT 47/40 (Bruker, Ettlingen, Germany). Infarct volume was calculated using ImageJ 1.44l (NIH, Bethesda, MD, USA) from the T2-weighted images.

In addition, infarct volume was determined by Nissl staining at later times: animals subjected to MCAO were killed 7 and 14 days after surgery and subjected to perfusion fixation with 4% p-formaldehyde in 0.1 M phosphate buffer (pH 7.4); brains were then frozen, serially sectioned at 40 μm, and stained with conventional histological Nissl (0.1% cresyl violet) staining. With the observer masked to the experimental conditions, the areas of the infarcted tissue (InfArea), the whole ipsilesional hemisphere (IpsArea), and the whole contralesional hemisphere (ContrArea) were delineated for each slice. Then, infarct volume, expressed as % of the hemisphere that is infarcted (%IH), was calculated as previously described [9] using the formula: %IH = InfVol/ContrVol*100 where InfVol (Infarcted Tissue Volume) = ∑InfArea_i_/SwellingIndex_i_, ContrVol (Contralesional Hemisphere Volume) = ∑ContrArea_i_, and SwellingIndex_i_ = IpsArea_i_/ContrArea_i_.

### Immunofluorescence studies

Animals (*n* = 6 to 7 for each group) were killed 7 or 14 days after MCAO by pentobarbital overdose followed by transcardiac perfusion through the left ventricle with 0.1 M phosphate buffer as a vascular rinse followed by a fixing solution containing 4% p-formaldehyde in 0.1 M phosphate buffer (pH 7.4). Brains were removed, postfixed overnight, and placed in 30% sucrose for 48 h. Coronal series sections (40 μm) were cut on a freezing microtome (Leica SM2000R, Leica Microsystems GmbH, Wetzlar, Germany) and stored in cryoprotective solution. Double-label immunofluorescence was performed on free-floating sections and incubated overnight at 4°C with the primary antibodies rat anti-BrdU (1:100; ABD Serotec, Bio-Rad Lab. Inc., Hercules, CA, USA), rabbit anti-Iba1 (1:500; Wako, Osaka, Japan), goat anti-doublecortin (DCX; 1:500; Santa Cruz Biotech, Heidelberg, Germany), mouse anti-NeuN (1:200 MAB 377; Millipore, Billerica, MA, USA), and rabbit anti-glutamic acid decarboxylase 67 (GAD67; 1:200; Abcam pic). After incubating with the primary antibody, sections were washed and incubated with the appropriate fluorescent secondary antibody: goat anti-rabbit biotin and horse anti-goat biotin (Vector Lab, Peterborough, UK) in combination with Alexa488 streptavidin (Molecular Probes, Life Tech, Madrid, Spain), Alexa647 (Invitrogen SA, Barcelona, Spain), and donkey Cy3 anti-rat/anti-mouse (Jackson Immunoresearch, Suffolk, UK). Controls performed in parallel without primary antibodies showed very low levels of nonspecific staining.

For the immunofluorescence study of *apoptosis* by determination of caspase-3 positive cells, sections were pre-treated using heat-mediated antigen retrieval with sodium citrate buffer (10 mM, pH6, in 0.05% Tween 20) for 40 minutes and 100°C and then incubated overnight with antibody caspase-3 (1:15, Enzo). Next day, sections were washed and incubated with goat anti-rabbit biotin secondary antibodies (Vector Laboratories, Peterborough, UK) in combination with Alexa488 streptavidin (Molecular Probes; Life Technologies, Madrid, Spain). Confocal images were taken from three correlative sections. Stacks at 20× of the infarct zone were obtained and analyzed with the ImageJ 1.44l software.

### Cell quantification and optical density on confocal images

For the study of neuroblasts (DCX^+^ cells), immunofluorescence images were obtained in a blinded manner from four correlative slices of each brain. Stacks at 20× of the subventricular zone (SVZ; Z1) were obtained. In addition, two adjacent images (Z2 and Z3) along the corpus callosum were also obtained. With the ImageJ v.1.44l software (NIH, Bethesda, MD, USA), each image was converted into a binary image and the integrated density (IntDen) was calculated. The IntDen is a calculus of the mean stained area times the intensity of stain in each pixel in the area and indicates the total amount of stained material in that area.

For neuronal differentiation (BrdU^+^/NeuN^+^ cells), immunofluorescence images were taken from five correlative sections beginning in 1.70 mm from the bregma (until 0.02 mm). The images were taken at 40× and spaced 400 μm from each other. The entire top of the cortex was traced and 800 μm below the stroke, using as boundaries of the corpus callosum and the end of the cortex, by analyzing a total of about 16 to 18 images per hemisphere and section.

The number of BrdU^+^ and BrdU^+^/Iba^+^ cells was quantified on digitalized confocal images captured from six serial 40-μm sections spaced 0.32 mm apart (two fields of view per section; Zeiss LSM 710) in ipsi- and contralateral cortices. Three-dimensional colocalization of protein markers was validated using confocal z-stacks.

### Unbiased stereology

The total volume of the dorsolateral striatal extension of the subventricular zone (SVZ) was estimated by the application of the Cavalieri’s principle on seven serial sections per brain (40-μm thickness, 0.32 mm apart) (bregma 1.70 mm to -0.54 mm). The morphological criteria used for the consistent delineation of the SVZ are described elsewhere [10]. Stereological estimation of the total number of BrdU^+^ cells in the dorsolateral extension of the SVZ was performed using the optical fractionator method [11]. The specific parameters used for stereological sampling and quantification of BrdU^+^ cells are described in [7].

### Brain dissociation and cell suspensions analysis by flow cytometry

Brain cell suspensions were prepared as described in [12,13]. Briefly, 1 and 2 days after MCAO, mice brain was rapidly removed. Cortex and subventricular zones were dissected with a scalpel and placed into 15 mL of ice-cold PBS.

In the case of *SVZ*, cells were dissociated in a single cell suspension using an enzymatic dissociation kit (neural tissue dissociation kit) containing papain with subsequent mechanical digestion with gentleMACS™ Dissociator (Miltenyi Biotec, Madrid, Spain) according to the manufacturer’s instructions. Cell suspensions were filtered on 40-μm nylon mesh strainers and centrifuged at 300 *g* for 10 min at room temperature. Pellets were resuspended on 200 μl of 5% BSA in PBS with Fc Block reagent (Miltenyi, Biotec, Madrid, Spain). Cell suspensions were filtered on 40-μm nylon mesh strainers and centrifuged at 300 g for 10 min at room temperature. Pellets were resuspended on 200 μl of 5% BSA in PBS with Fc Block reagent (Miltenyi). Cell suspensions were co-labeled with antibodies for neural stem cell marker PE anti-Prominin1/CD133 (Miltenyi Biotec, Madrid, Spain), primitive neural stem cell marker PerCP-Cy 5.5 anti-Nestin (BD Pharmingen, San Jose, CA, USA), a astrocytes like-stem cell marker rat anti-GFAP (Invitrogen; with donkey anti-rat Alexa Fluor 647, Jackson Immunoresearch) and two markers for proliferative progenitors, FITC-labeled epidermal growth factor receptor (FITC anti-EGFR; Invitrogen, Life Tech, Madrid, Spain), and mouse anti-Mash1 (BD Pharmingen, San Jose, CA, USA; and goat anti-mouse Alexa Fluor 647, Jackson Immunoresearch). For labeling the cytoplasmic protein nestin, GFAP and Mash1, suspensions were mixed with permeabilization solution (BD Cytofix/Cytoperm Kit, BD Biosciences, Madrid, Spain) for 20 min at 4°C and stained overnight.

In the case of *cortex*, cells were dissociated in a single cell suspension using a gentleMACS™ Dissociator (Miltenyi) according to manufacturer’s instructions. Cell suspension was filtered on 50-μm nylon mesh strainers (BD Biosciences) and centrifuged at 300 *g* for 10 min. Pellets were resuspended in 3 mL of 50% Percoll and overlaid on the top of a gradient containing 3 mL of 30% of Percoll. The gradient was centrifuged at 500 *g* for 20 min at room temperature. Cells were collected from the 30% to 50% interface and resuspended on 200 μl of 2.5% BSA in PBS with Fc Block reagent (Miltenyi). Cell suspensions were incubated with conjugated antibodies CD11b-PerCP, CD45-PE (Miltenyi, Biotec, Madrid, Spain), and Ly-6G-APC (Biolegend, San Diego, CA, USA). After membrane staining, cells were mixed with permeabilization solution (BD Cytofix/Cytoperm Kit, BD Biosciences, Madrid, Spain) for 20 min at 4°C and stained overnight to proceed to the staining of intracellular Ym1 (1:100; StemCell Technologies Inc., Vancouver, British Columbia, Canada) and CCR2 (Abcam) followed by incubation with immunofluorescent anti-rabbit-Alexa 488 and anti-goat Alexa 647 (Life technologies, Madrid, Spain).

Stained cells from cortex and SVZ were washed and resuspended in 300 μl of FACS Flow (BD Pharmingen, San Jose, CA, USA), and the whole suspension was acquired using a FACSCalibur flow cytometer with CellQuest software (BD Pharmingen, San Jose, CA, USA). Isotype controls (Miltenyi, Biotec, Madrid, Spain) were used in parallel.

### Blood cells characterization by flow cytometry

Peripheral blood cells were incubated for 10 min with a standard ammonium chloride lysing solution. Samples were washed twice with phosphate-albumin buffer (PAB; 0.0455% sodium azide and 0.1% bovine serum albumin) and resuspended in PAB and mouse Fc Block (1:500; BD Pharmingen, San Jose, CA, USA). Of cell suspension, 200 μl was incubated with conjugated antibodies CD11b-FITC clone M1/70 and Ly6C-PE (Miltenyi Biotec, Madrid, Spain) and Gr1-APC (RD system, Minneapolis, MN, USA). The stained cells were washed and resuspended in 300 μl of FACS Flow (BD Pharmingen, San Jose, CA, USA). All the events were acquired using a FACSCalibur flow cytometer with CellQuest software (BD Pharmingen, San Jose, CA, USA). Granulocytes were identified by forward and side scatter analysis and confirmed by their expression of CD11b and Gr1.

### Statistical analysis

Data were expressed as mean ± SD. Comparisons between groups were performed using unpaired Student’s *t*-test or one-way ANOVA with the Student-Newman Keuls *post hoc* test for multiple comparisons. Comparisons of semiquantitative scores were analyzed with nonparametric Mann-Whitney *U*-test. Linear association between two variables was determined by the Pearson correlation coefficient. Differences were considered significant at *P* < 0.05.

## Results

### Effect of aging on infarct size

**Figure 1:**
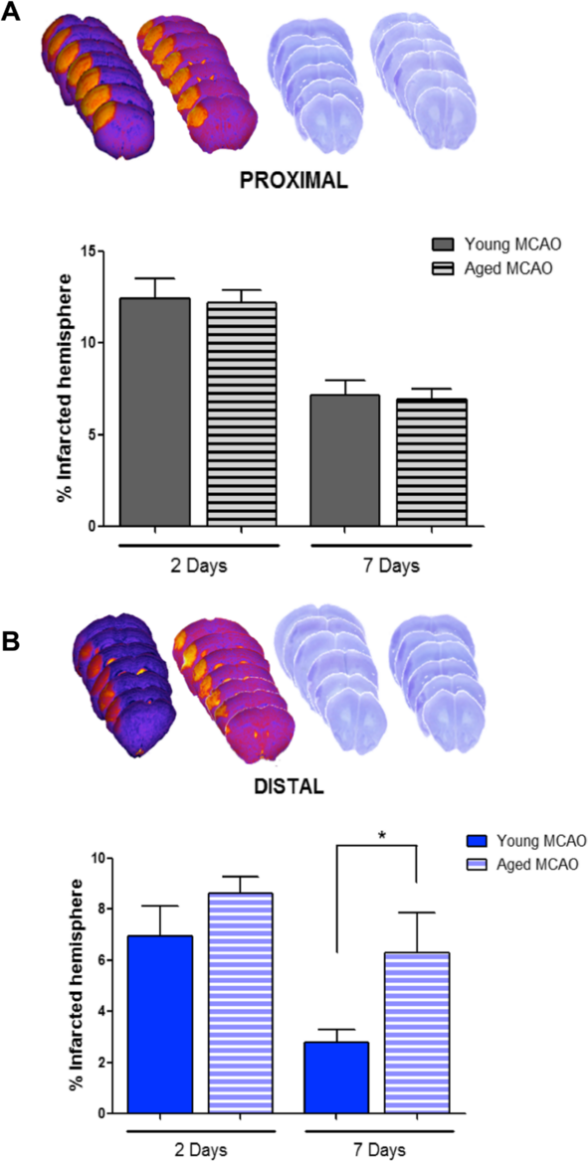
Effect of aging on infarct development. Infarct size was determined by MRI (2 days) and Nissl (7 days) after MCAO in young (2 to 3 months) and aged (13 to 14 months) mice after proximal **(A)** and distal **(B)** occlusions (see Methods). Data are mean ± SD, *n* = 8, *P < 0.05. MCAO, middle cerebral artery occlusion.

First, permanent MCAO was performed on young and aged C57BL/10 J mice either by distal or proximal occlusion of the MCA, and the size of the infarct lesion was determined 2 and 7 days later. Infarct volume at 2 days was not affected by aging in both types of MCAO studied (proximal and distal; Figure 1A, B). However, whereas the size of the lesion equally regressed after proximal occlusion in both young and aged animals at day 7 (Figure 1A), this effect was not observed in the group of aged animals with distal occlusion (Figure 1B; *P* < 0.05). Therefore, we decided to use the groups with distal occlusion for subsequent studies.

### Effect of aging on microglial proliferation

**Figure 2:**
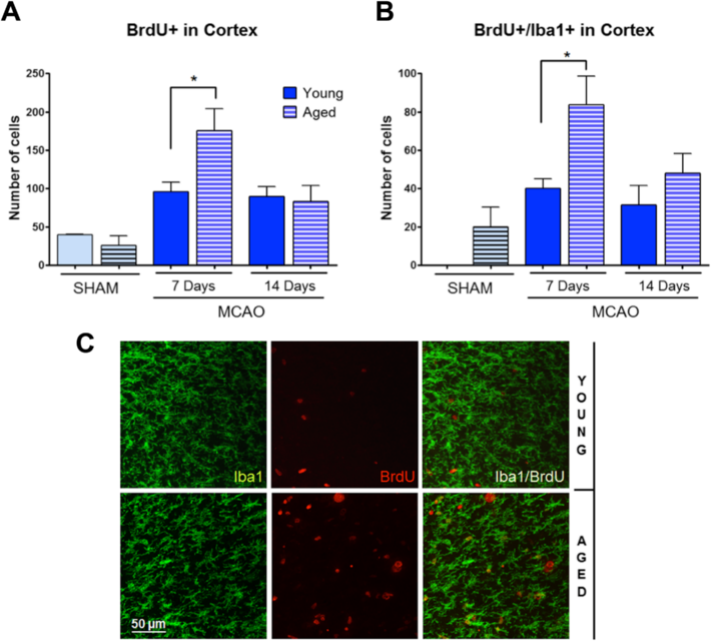
Effect of aging on microglial proliferation and cell infiltration. Number of BrdU^+^ **(A)** and BrdU^+^/Iba1^+^ **(B)** cells in the peri-infarct area (cortex) at 7 and 14 days after MCAO in young and aged mice. The number of BrdU^+^ and BrdU^+^/Iba1^+^ cells (microglial/macrophages cells) was quantified on digitalized confocal images (see Methods). Representative images of Iba1^+^ cells in the ipsilesional peri-infarct hemispheres in young and aged mice. Scale bar = 50 μm **(C)**. MCAO, middle cerebral artery occlusion.

As expected, ischemia induced microglial proliferation in the ipsilesional cortex when compared with the sham group, as demonstrated by an increased number in both BrdU^+^ (Figure 2A) and BrdU^+^/Iba1^+^ (Figure 2B) cells in the peri-infarct area. Importantly, brains of aged animals had a higher number of BrdU^+^ cells and BrdU/Iba1^+^ cells in the ischemic boundary when compared with the young mice 7 days after MCAO (40% BrdU^+^ and 60% BrdU^+^/Iba1^+^*P* < 0.05 aged vs. young; Figure 2A-C).

### Effect of aging on cell infiltration

**Figure 3:**
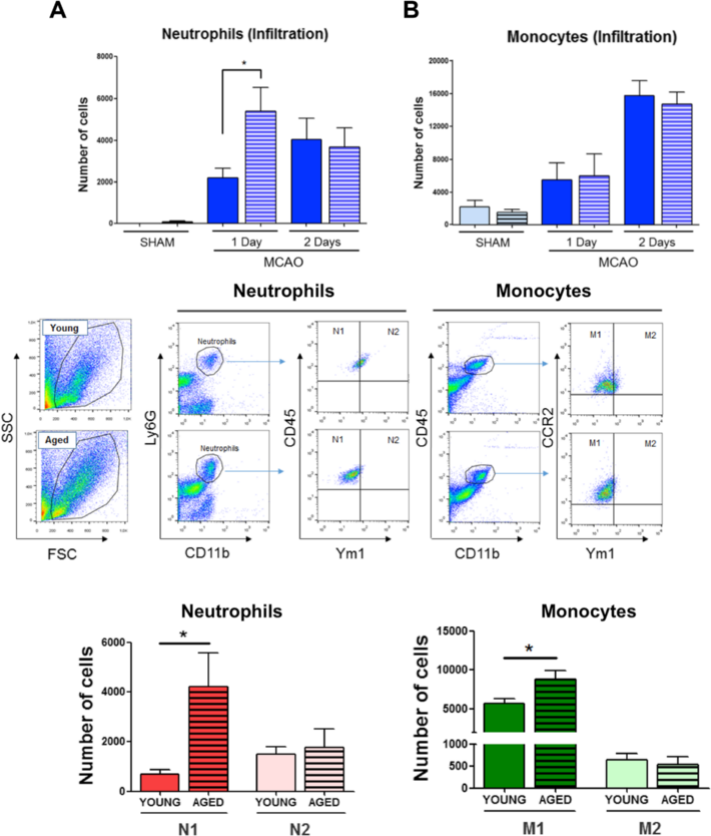
Effect of aging on cell infiltration. Flow cytometric analysis of brain neutrophils and monocytes in sham or ischemic mice brain 1 and 2 days after ischemia in young and aged mice **(A, B)**. Upper: quantification of total neutrophils and monocytes. Right Lower: quantification of N1 (Ym1^−^) and N2 (Ym1^+^) neutrophils (Cd11b^+^, Ly6G^hi^, CD45^hi^). Left Lower: quantification of M1 (Ym1^−^) and M2 (Ym1^+^) monocytes (Cd11b^+^, CD45^hi^, CCR2^+^). Data are mean ± SD, *n* = 6 to 8 in each group. *P* < 0.05.

The number of infiltrated neutrophils was higher in aged than in young mice analyzed by flow cytometry 1 day after stroke (Figure 3A). We did not find any differences on monocyte infiltration in both groups studied (Figure 3B). Therefore, we studied M1/M2 infiltrated monocytes and N1/N2 infiltrated neutrophils populations 1 day after stroke, demonstrating that aged animals not only had a higher number of infiltrated M1 monocytes (Cd11b^+^, CD45^hi^, CCR2^+^, Ym1^−^ cells, Figure 3B and plot) but also of N1 neutrophils (Cd11b^+^, Ly6G^hi^, CD45^hi^, Ym1^−^ cells, Figure 3A and plot) than young animals (*P* < 0.05 young vs. aged; Figure 3A, B).

Finally, we also explored the different populations of circulating leukocytes (neutrophils and monocytes) by flow cytometry, but we did not detect any differences between young and aged mice (data not shown).

### Effect of aging on SVZ cell proliferation

**Figure 4:**
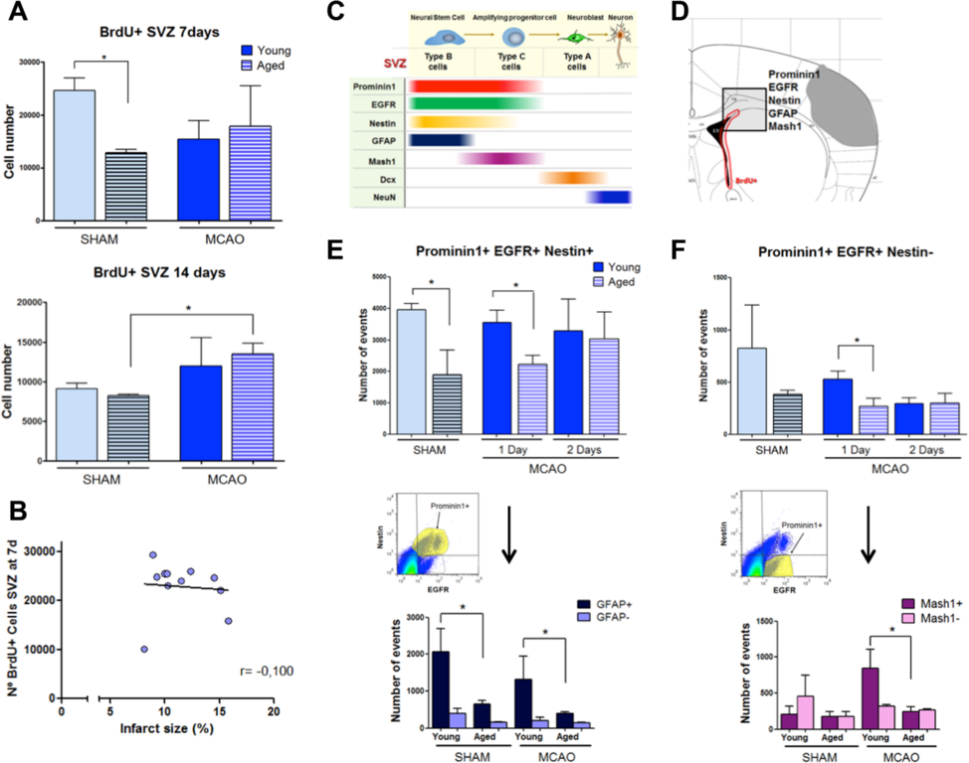
Effect of aging on SVZ cell proliferation. **(A)** Number of BrdU+ cells in the SVZ at 7 and 14 days after MCAO in young and aged mice. Data are mean ± SD, *n* = 6 to 8. **(B)** Correlation between number of BrdU+ cells in SVZ at 7 days and infarct size from aged mice (*n* = 10). **(C)** Diagram with SVZ cell markers. **(D)** Scheme. **(E)** Cytometric quantification of prominin-1^+^/EGFR^+^/nestin^+^ cell population (right upper) and of type-B cells (GFAP^+^ cells; right lower). **(F)** Quantification of prominin-1^+^/EGFR^+^/nestin^−^ cell population (left upper) and of type-C cells (Mash1+ cells; left lower). SVZ cells were obtained after MCAO from young and aged mice. Cell suspensions were labeled with antibodies for the different markers and analyzed by flow cytometry (see Methods for details). Data are mean ± SD, *n* = 6 to 8, *P < 0.05. MCAO, middle cerebral artery occlusion; EGFR, epidermal growth factor receptor; GFAP, glial fibrillary acidic protein; SVZ, subventricular zone.

In order to ascertain the effect of aging on SVZ cell proliferation, we first studied the number of BrdU^+^ cells in this area 7 and 14 days after MCAO, after a pulse of BrdU at days 5 to 6. Exposure to distal MCAO did not significantly affect the number of BrdU^+^ cells in the SVZ, either in young or in aged mice, when studied 7 days after MCAO. However, in aged animals, MCAO caused an increase of the BrdU^+^ cells in the SVZ at 14 days after injury (Figure 4A, *P* < 0.05). In addition, aged sham-operated animals showed a lower number of proliferative cells in the SVZ at 7 days (BrdU^+^ cells; Figure 4A, *P* < 0.05), as previously reported for physiological neurogenesis [14].

Since we have recently reported that SVZ cell proliferation depends on infarct size after stroke in young mice [8], we decided to study whether this effect is also observed in aged mice, by including an additional group of mice in which MCAO was performed proximally in order to obtain different lesion volumes. As expected, the groups with the proximal occlusion had significantly larger infarct volumes than the distal occlusion groups (Figure 1A). However, when we studied SVZ cell proliferation (BrdU^+^ cells) at day 7 after stroke in the groups of aged animals (proximal and distal occlusion), we did not find any correlation between infarct size and cell proliferation determined at the ipsilateral SVZ 7 days after injury (Figure 4B; *r* = -0.100, *n* = 10, *P* > 0.05) in contrast with the positive correlation found in young mice [8].

In order to confirm the effect of aging on SVZ proliferation and to establish the nature of the cells affected in the SVZ, we characterized by flow cytometry the different cell types present in this area at earlier times, 1 and 2 days after distal MCAO, in young and aged mice. Our results show the presence of two major cell populations, as we have previously reported [8]: a population of prominin-1^+^/EGFR^+^/nestin^+^ cells in which approximately 90% are GFAP^+^ cells, corresponding to the primary neural stem cells (astrocyte-like type-B cells) and a second population of prominin-1^+^/EGFR^+^/nestin^−^ cells, consistent with the transit-amplifying cells (type-C cells), in which about 50% are Mash1^+^ (Figure 4F), corresponding to the type C. Importantly, we found that aged mice showed a smaller number of type-B cells in sham animals and also 1 day after stroke (Figure 4E; young MCAO vs. aged MCAO; *P* < 0.05), suggesting a global inhibitory role of aging in the proliferation of type-B cells.

In contrast, in the prominin-1^+^/EGFR^+^/nestin^−^ cell subset (type-C cells), we have found a specific and marked increase of Mash1^+^ cells only after MCAO and in young but not in aged mice (Figure 4F; young MCAO vs. aged MCAO; *P* < 0.05). Therefore, aging inhibits proliferation of type-B and C cells after stroke.

Finally, in order to discard that the decreased cell number found in the group of aged animals was not due to increased apoptosis [6], we determined apoptosis (caspase-3 positive cells) in both the SVZ and in the cortex from young and aged animals. We did not find any differences between both groups of animals at both tissues and time-points (7 and 14 days after stroke) studied (data not shown).

### Effect of aging on neuroblast migration toward the injured area

**Figure 5:**
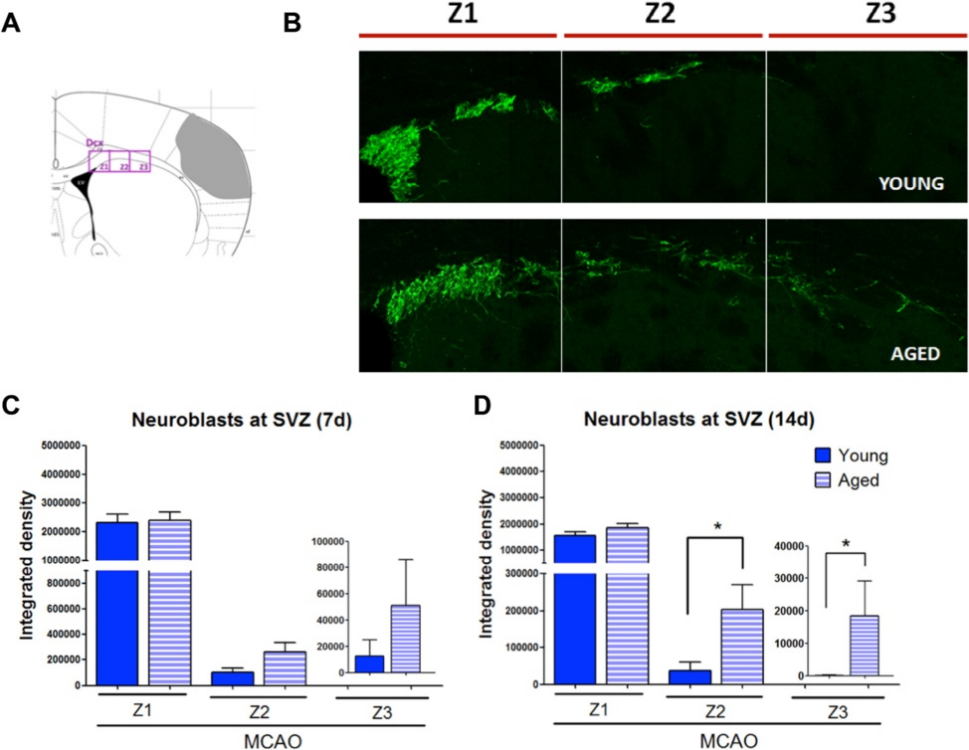
Effect of aging on neuroblast migration. **(A)** Scheme, **(B)** representative images, and **(C, D)** number of neuroblasts (doublecortin^+^ DCX^+^ cells) in three migratory zones (Z1, Z2, Z3) at 7 and 14 days after MCAO in young and aged mice. Migratory zones were established from the SVZ (Z1) through the corpus callosum (Z2) toward the damaged area (Z3) (see Methods). Data are mean ± SD, *n* = 6 to 8, *P < 0.05. Scale bar = 200 μm. MCAO, middle cerebral artery occlusion.

We next explored the effect of aging on the neuroblast population (type-A cells) in 3 migratory zones ranging from the SVZ (Z1) through the corpus callosum (Z2) toward the damaged area (Z3), 7 and 14 days after the ischemic insult. We did not find any difference in the density of neuroblasts (DCX^+^ cells) when young-adult and aged mice were compared 7 days after MCAO (Figure 5C). Importantly, 14 days after MCAO, the density of DCX^+^ cells in Z2 and Z3 was higher in aged than in young mice (Figure 5B, D; *P* < 0.05). When each group was analyzed separately, we found that DCX^+^ cells in Z2 and Z3 decreased from 7 to 14 days in the young but not in the aged group, suggesting impairment in migration caused by aging.

### Effect of aging on the number of new neurons in the peri-infarct cortex

**Figure 6:**
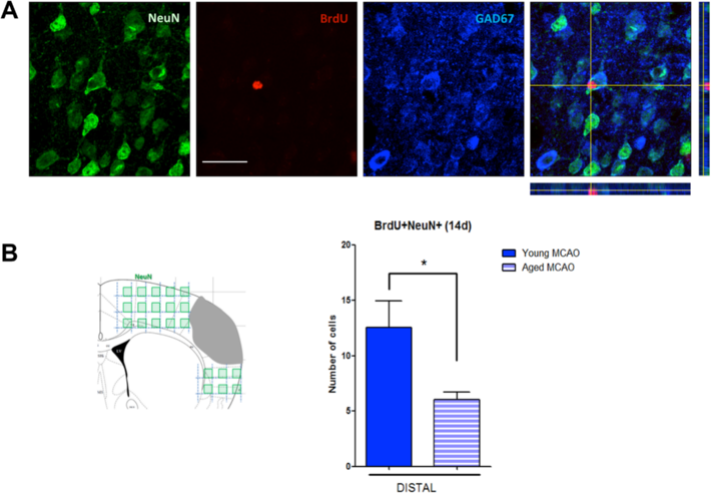
Effect of aging on the number of new neurons in the peri-infarct cortex. **(A)** Representative images and **(B)** number of adult interneurons (BrdU^+^/NeuN^+^/GAD67^+^ cells) in the ipsilesional cortex at 14 days after MCAO in young and aged mice. Data are mean ± SD, *n* = 6-8, *P < 0.05. Scale bar = 25 μm.

Finally, we studied the number of mature differentiated interneurons (BrdU^+^/NeuN^+^/GAD67^+^ cells) in the ipsilesional cortex 14 days after the ischemic insult. Interestingly, we found that the number of BrdU^+^/NeuN^+^/GAD67^+^ cells in the peri-infarct cortex was significantly lower in aged than in young mice 14 days after MCAO (Figure 6A, B; young MCAO vs. aged MCAO, *P* < 0.05). No new neurons (BrdU^+^/NeuN^+^) were found in the cortex of sham-operated mice.

## Discussion

In this study, we have explored the role of aging on neurogenesis after stroke by using a permanent model of focal ischemia in young and aged mice. Our data show that (i) aging increases microglial proliferation in the ischemic boundary and the number of M1 monocytes and N1 neutrophils, whilst decreasing the alternative M2 and N2 phenotypes; (ii) cell proliferation (number of BrdU^+^ cells) at the SVZ is dependent on infarct size after stroke in young but not in aged animals; (iii) aging inhibits SVZ cell proliferation, as shown by a decreased number of both type-B cells (prominin-1^+^/EGFR^+^/nestin^+^/GFAP^+^ cells) in both sham and ischemic brains and of type-C cells (prominin-1^+^/EGFR^+^/nestin^−^/Mash1^+^ cells) at day 1 after stroke but not in sham mice; (iv) aging inhibits migration of neuroblasts as shown by an accumulation of neuroblasts (DCX^+^ cells) in Z2 and Z3 migratory zones at 14 days after injury; and (v) consistently, aged mice present a smaller number of differentiated interneurons (NeuN^+^/BrdU^+^/GAD67^+^ cells) in the cortical peri-infarct area at 14 days after stroke. We have also found that for small cortical infarcts (obtained by distal occlusion), lesion does not regress in aged mice 7 days after injury whilst it does in young animals.

Indeed, after a distal occlusion, the size of the lesion does not regress in aged mice at 7 days after injury as it does in young mice, showing larger infarcts at this time. A possible justification to explain the regression in small but not large infarcts could lie on the fact that small infarcts are more amenable to modulation by different mechanisms such as those processes that underlie the evolution of ischemic penumbra at shorter times, the response of resident microglia and the degree of infiltration of peripheral cells. Indeed as we commented below, both microglia proliferation and neutrophil infiltration are increased in aged mice using this model, and both could underlie the smaller regression found. Previous reports showed that aged rodents develop larger infarct volumes than young animals when determined at early times poststroke [15]. However, we have found this difference at later times (7 days after injury) when they did not find any significant effect. Factors such as the ischemic model used, degree of aging of the animals, species, or strain of animals might explain this apparent controversy [1,15,16].

Several issues have been described to obstruct the recovery of the lesion in aged animals, including the so-called ‘inflamm-aging’ state [2] found in aged animals. Indeed, we have found that aged animals show a higher proliferation of microglia in the ipsilateral cortex as demonstrated by an increase in BrdU^+^ and BrdU^+^/Iba1^+^ cells 7 days after ischemia when compared with young mice. Our results support the existence of an activated basal state (inflamm-aging) and are in agreement with previous reports describing that the number of BrdU^+^ cells (45% of them corresponding to reactive microglia) in the infarcted hemisphere of aged rats at days 3 and 7 vastly exceed those of young rats [15,17]. Consistently, it has been described that basal mRNA expression of CD11b and Iba1, markers of activated microglia, is higher in the aged than in the young brain after stroke [4]. Importantly, we have also found a higher number of infiltrated neutrophils in aged animals, as previously demonstrated [18]. In this context, our data also show that aging enhances the number of M1 monocytes and N1 neutrophils, phenotypes associated to a pro-inflammatory state. Our results are in agreement with those demonstrating that aging enhances classical M1 activation, mitigating alternative M2 activation [19]. The evidence in the literature on N2 neutrophils is still scarce, as this phenotype was only recently described [20]. N2 neutrophils, similarly to M2 monocytes, have been implicated in neuroprotection by increasing neutrophil clearance and resolving inflammation [20].

We have also found that, in aged mice and under ischemic conditions, SVZ cell proliferation (BrdU^+^ cells) 7 days after the injury does not depend on infarct size as shown by the negative correlation found. We recently published that in young animals there is a correlation between SVZ cell proliferation and infarct size [8]. The reduced neurogenesis under physiological conditions and after stroke that we have found in aged animals might explain the lack of correlation found. In fact, our data in sham-aged animals show that physiological neurogenesis is reduced in the brains of aged rodents, with a decrease in the number of BrdU^+^ cells (proliferating cells) and type-B cells (prominin-1^+^/EGFR^+^/nestin^+^/GFAP^+^ cells), in agreement with previous results (for review, see [5]). Interestingly, we have found that, after stroke, aging not only decreases the number of astrocyte-like type B (in both sham and MCAO groups) but also of type-C cells (prominin-1^+^/EGFR^+^/nestin^−^/Mash1^+^ cells) in the SVZ, in this case, only after injury. Altogether, our results indicate that aging affects neural progenitors, although the effect is different in each cell population either in control (sham) or in ischemic conditions. We have also demonstrated that the decreased proliferation found in the group of aged animals is not due to increased apoptosis as previously reported [6], at least at the time studied. To our knowledge, this is the first demonstration that after stroke, aging inhibits the proliferation of type-B and C cells in the SVZ. Our results are in agreement with those from Jin *et al*. showing that stroke-induced neurogenesis is preserved but reduced in the aged rodent brain [21].

Furthermore, we have shown that aging delays or blocks the neuroblast migration process from the SVZ toward the damaged area. In our study, we examined the neuroblast population (type-A cells; DCX^+^) in three migratory zones ranging from the SVZ (Z1) through the corpus callosum (Z2) toward the damaged area (Z3), at two different times, 7 and 14 days after the ischemic insult. Two weeks after the infarct, we found that the density of neuroblasts at the Z2 and Z3 zones, despite a decreased proliferation at the SVZ, increased in aged mice when compared with those from young animals, suggesting that aging hampered the migration process resulting in an accumulation of these cells. Several factors might be involved in the altered migration process found in aged animals. Although we did not find any correlation between microglia and neuroblast migration, we cannot discard that the intense inflammatory reaction (inflamm-aging) that we have found in aged mice might contribute to a rapid formation of growth-inhibiting scar tissue in the infarcted area that might impair migration as reported [3,17]. In addition, it has been described that factors involved in the migration process such as EGF [22] and VEGF [23] are downregulated or inhibited during aging [24,25].

Finally, our data show that, in young animals, stroke induces the appearance of new neurons close to the damaged area, which are interneurons (GAD67^+^ cells). Although the number found appears relatively low, it is likely to be remarkably underestimated, as the figures reported only represent the fraction of cells that reach the infarct and differentiate into neurons among those that proliferated at days 5 and 6 after the ischemic injury, when the BrdU pulse was applied. Importantly, we have found that aged mice show a substantially decreased neurogenesis at the cortical area at 14 after stroke, in accordance with the suppression of the migratory process found. Aging may therefore explain recent evidence reporting that neurogenesis is negligible in the human neocortex after ischemic stroke in aged patients (average age of 70; [26].

## Conclusions

In summary, we have confirmed that in aged mice, stroke-induced neurogenesis is maintained but reduced. Importantly, we are now demonstrating that aging affects proliferation of specific populations of neural progenitor cells, blocks migration of neuroblasts to the damaged area, and decreases the number of new interneurons in the cortical area. Therefore, the search for the mechanisms that underlie this process might have important therapeutic implications in old patients with stroke in order to increase the different processes of neurogenesis. Furthermore, our results showing different effects depending on age of animals, strongly remark the importance of using aged animals for the study of stroke.

## Abbreviations

BrdU: 5-Bromo-2′-deoxyuridine
MCA: middle cerebral artery
MCAO: middle cerebral artery occlusion
SVZ: subventricular zone
